# Complementarities between algorithmic and human decision-making: The case of antibiotic prescribing

**DOI:** 10.1007/s11129-024-09284-1

**Published:** 2024-07-05

**Authors:** Michael Allan Ribers, Hannes Ullrich

**Affiliations:** 1 Department of Economics, University of Copenhagen, Copenhagen, Denmark; 2 DIW Berlin, Department Firms and Markets, Berlin, Germany

**Keywords:** Human-machine complementarity, Machine learning, Antibiotic resistance, Antibiotic prescribing, C53, D83, I18, I19, L2, M15

## Abstract

Artificial Intelligence has the potential to improve human decisions in complex environments, but its effectiveness can remain limited if humans hold context-specific private information. Using the empirical example of antibiotic prescribing for urinary tract infections, we show that full automation of prescribing fails to improve on physician decisions. Instead, optimally delegating a share of decisions to physicians, where they possess private diagnostic information, effectively utilizes the complementarity between algorithmic and human decisions. Combining physician and algorithmic decisions can achieve a reduction in inefficient overprescribing of antibiotics by 20.3 percent.

## Introduction

Professionals and domain experts frequently make costly decisions under time pressure and with limited information, often processed with a host of biases (Thaler & Sunstein, 2009; Kahneman et al., 2021). Advances in computing power and rapidly increasing data availability have provided new potential solutions for high-stakes problems with prediction at their core (Kleinberg et al., 2015). Hopes are high that machine learning can help improve human decision-making by offering a systematic prediction of the ground truth and guiding optimal decisions. Yet, humans often hold abstract, context-specific information that may be difficult to assess using machine learning (Autor, 2015). Employers observe candidates’ soft skills in job interviews, judges learn about defendants’ personalities in face-to-face questioning, and physicians observe patients’ ailments with potentially complex symptoms. Empirical evidence on the relevance and nature of complementarities between data-driven and human decisions is scarce but key for guiding policy-making in response to the economic transformation induced by artificial intelligence.

In this paper, we provide such evidence for a salient case in health care. Antibiotic resistance is one of the greatest threats to global health (WHO, 2012, 2014).^1^ Because human antibiotic consumption is considered the main driver of antibiotic resistance, reducing the use of antibiotics is a prime policy concern (Goossens et al., 2005; Adda, 2020). The decision to use an antibiotic involves a prediction task in determining the cause of a patient’s illness. Physicians collect and interpret clinical facts including symptoms, point-of-care test results, and maybe patients’ background and medical data, requiring human judgment and curiosity. On the other hand, machine learning has shown to be an effective method to elicit predictive information from large-scale data (Agrawal et al., 2018; Athey, 2018). It can make use of systematic patterns in data collected across patients and healthcare providers such as electronic health records, administrative data, and genomics databases. Yet, machine learning applications face challenges when crucial, treatment-relevant physician information is not encoded in a standardized way and not easily combined with other data. Integrating physician decisions in algorithmic rules may provide a solution (Agarwal et al., 2023).

The treatment of urinary tract infections (UTI) in primary care, a leading cause for human antibiotic use (Grigoryan et al., 2014), provides a unique setting to study the potential to reduce antibiotic use with the help of machine learning-predicted risk. An accurate diagnostic for UTI can only be provided by analysis of urine samples in a microbiological laboratory outside of primary care clinics. These laboratory test results arrive with a delay of several days, corresponding to nearly a full course of antibiotic treatment. Thus, at initial consultations, physicians must decide under uncertainty whether to prescribe an antibiotic or delay treatment until the test result is known.

Crucially, because *ex post* positive and negative laboratory results, as well as the initial treatment decisions, are observed, prescription decisions can be evaluated based on the true outcome. Hence, we avoid the common selective labels problem for the decision of interest (Lakkaraju et al., 2017; Kleinberg et al., 2018a). To achieve this, we restrict our analysis to consultations at which a laboratory test is acquired. While this restriction may limit the external validity of the quantitative results, which we inspect in robustness checks, our setting provides a unique lens to measure complementarities between physician and prediction-based decisions.

We first apply a machine learning algorithm, XGBoost, to high-dimensional, administrative data from Denmark to predict the risk of bacterial presence for 48,406 initial consultations. The outcome is a binary variable indicating when bacteria are isolated in a patient’s urine sample in the laboratory. The prediction model includes patients’ historical medical outpatient claims, antibiotic prescriptions, microbiological test results, personal characteristics such as gender, age, employment information, education, income, civil status, clinic identifiers, past test yield, time indicators, and more. XGBoost predicts bacterial infections out-of-sample with an area under the ROC curve (AUC) of 0.72. This prediction quality is comparable with values in the literature, for example, Mullainathan and Obermeyer (2022) with 0.69 for heart attacks, Kleinberg et al. (2018a) with 0.707 for risk of recidivism, and between 0.56 and 0.83 for predicting antibiotic resistance conditional on the presence of bacteria and antibiotic prescription in Yelin et al. (2019) and Kanjilal et al. (2020).

The policy problem we analyze involves a trade-off between the social cost of prescribing, i.e. promoting resistance, and the health benefits of antibiotic treatment. Using an objective function that reflects this trade-off, we consider policies that reassign antibiotic treatment based on risk predictions to reduce antibiotic use. Observing that physicians make the fewest errors relative to machine learning in intermediate ranges of predicted risk, we evaluate rules that delay prescriptions until test results are available for low predicted risk, prescribe an antibiotic instantly for high predicted risk, and delegate decisions to physicians for intermediate predicted risk.

Applying this policy, assuming physicians comply, antibiotic use can be reduced by 8.1 percent without reducing the number of treatments to patients suffering from a UTI. The policy can reduce overprescribing, prescriptions to non-bacterial cases, by 20.3 percent. In 47.2 percent of consultations, the decision would be made by the prediction-based rule, overturning 15.0 percent of the observed decisions made by physicians. We find that only decision rules that combine machine learning and human decisions improve outcomes, even with the rich individual-specific data in this setting.

We document that including human decisions in the algorithm is optimal when physicians contribute important diagnostic information not encoded in data. To quantify this contribution, we compute the difference between machine learning prediction error and physician decision error. This informational advantage of physicians over machine learning is largest at intermediate ranges of predicted risk and negative at low and high predicted risk. Correlating this measure with point-of-care diagnostic claims, we find that physicians’ informational advantage is largest where the use of such diagnostics is highest. Hence, physicians acquire and interpret important information at the point of care that is not available to the machine learning algorithm. While information is increasingly encoded for machine learning, the human informational advantage needs to be quantified to identify settings in which complementarities exist.

The type of administrative data we employ has been shown to provide similar prediction quality as when electronic health record data are used, even though they likely contain richer context-specific information (Zeltzer et al., 2019). Our findings indicate the value of combining administrative data with context-specific information collected by human experts. Ideally, the two data sources would be used together but combining them has shown to be a difficult problem in practice for a multitude of technical and legal reasons (Hsu et al., 2020). Integrating physician decisions, which carries much of the human-acquired information, can be a promising and pragmatic way to move forward.

We contribute to a growing literature considering prediction problems in management and policy (Kleinberg et al., 2015). Existing work has studied the potential for machine learning to improve decisions such as for crime prevention programs (Chandler et al., 2011), hygiene inspections (Kang et al., 2013), worker productivity (Chalfin et al., 2016), C-sections (Currie & MacLeod, 2017), tax rebate programs (Andini et al., 2018), opioid prescriptions (Hastings et al., 2020), financial stock analysis (Cao et al., 2021), and testing for heart attacks (Mullainathan & Obermeyer, 2022). Also focusing on UTI treatment, Ribers and Ullrich (2023) estimate the distribution of physicians’ skills and preferences determining antibiotic prescribing decisions and Huang et al. (2022) quantify the value of increasing the scope of data for prediction quality and policy outcomes. In this common and important health care context, we analyze how treatment decisions may be shared between human experts and an algorithm using simple policies.

Recent work has focused on algorithms as a substitute for human decisions but the question of whether data-driven models can complement human decisions has been investigated at least since Blattberg and Hoch (1990). As data sets have grown in scale and advances in computing have enabled increasingly flexible prediction models, the contribution of human intuition and information is becoming more nuanced. Valuable complementarities can arise if humans fill crucial remaining gaps where procedural expertise, subjective evaluations, highly flexible assessments, or domain-specific knowledge of rare events are required, commonly the case in abstract task-intensive occupations such as medical care (Autor, 2015). Contrary to Agrawal et al. (2018) and Agrawal et al. (2019), who focus on human judgment that is difficult to encode, we identify context-specific information humans acquire, which remains difficult to encode, as an important factor for policy design.

The paper is organized as follows. Section 2 provides background information on Danish primary care and UTI and Section 3 describes our data. Section 4 shows the results of the prediction algorithm. Section 5 presents the framework for prediction-based policies to improve antibiotic prescribing. Section 6 presents policy outcomes and Section 7 concludes.

## Institutional background and treatment of UTI

### Primary healthcare in Denmark

Denmark has several regulations that impact decision-making in primary care. General practitioners act as the primary gatekeepers in a universal and tax-financed single-payer health care system. Every person living in Denmark is allocated to a general practitioner by a list system within a fixed geographic radius around the home address. General practitioners work as privately owned businesses but all fees for services are collectively negotiated between the national union of general practitioners and the public health insurer. Physicians do not generate earnings by prescribing drugs to patients who have to purchase their prescriptions from local pharmacies. General practitioners are responsible for prescribing approximately 75 percent of the human-consumed systemic antibiotics in Denmark (Danish Ministry of Health, 2017). Pharmacies earn a fixed fee per processed prescription regardless of price or other drug attributes, for example, branded versus generic drugs. Prescription drugs are subsidized but patients co-pay a fraction of the list price. The Danish market for prescription drugs is highly regulated resulting in low and uniform prices for antibiotics nationwide, about 100 Danish Kroner (15 US Dollars) per complete treatment.

### Diagnosis and treatment of UTI

UTI are among the most common types of infections and a leading reason for antibiotic treatment in primary care (Grigoryan et al., 2014; Gupta et al., 2017). UTIs occur when bacteria, most often *Escherichia coli*, enter the urethra and infect the urinary tract, the bladder, or kidneys. Left untreated, they can lead to sepsis and death. The estimated costs to the health care system attributable to community-acquired UTI amount to $1.6-3.5 billion per year in the US alone (Foxman, 2002; Flores-Mireles et al., 2015). Once diagnosed, the use of antibiotics is indicated by clinical guidelines.^2^ In our setting, over 80 percent of UTI-indicated prescriptions are for pivmecillinam, belonging to the class of penicillins and recommended as a first-line antibiotic for UTI, or sulfamethizole.^3^

The prevalence of UTI is highest among women. Foxman (2002) reports that nearly half of all women experience at least one UTI in their lives. Many more subgroups are known to be at increased risk of UTI, such as children and the elderly, patients with certain conditions such as diabetes or immunodeficiency, or individuals with underlying urological abnormalities (Foxman, 2002). Many of such subgroups are identifiable in observable data using personal characteristics such as age and gender or past health care utilization and diagnoses.

UTI symptoms require medical attention. They include dysuria, urinary frequency, urgency, new-onset incontinence, and pain. Systemic signs of an infection such as fever, shivering, or systemic unwellness can also occur. Attributing symptoms to UTI is difficult as they are also associated with other conditions, e.g. sexually transmitted urethritis or vaginitis, noninfectious urethritis, early pyelonephritis, overactive bladder, benign prostatic hyperplasia, bladder or kidney stones, or even a bladder tumor (Wilson & Gaido, 2004; Gupta et al., 2017; Nik-Ahd et al., 2018; Holm et al., 2021). Less commonly, UTIs can also be caused by fungi or viruses. Notably, symptoms are difficult to encode systematically. For example, the assessment of “pain” requires contextual elicitation and judgment of its nature, severity, location, and chronology. Beyond symptoms, physicians may elicit contextual information, including behavioral factors, from speaking to patients. The quality and depth of recording this type of information can vary widely across clinics, patients, and time.

Point-of-care testing such as urinary dipstick and microscopy analysis provides diagnostic results at the consultation. Both types of diagnostics can have very low specificity, the true negative rate, as low as 0.41 or sensitivity, the true positive rate, as low as zero (Devillé et al., 2004; Wilson & Gaido, 2004; Chu & Lowder, 2018). Further analysis can be done by urine culture which takes about one day. Finally, samples can be sent to a hospital laboratory for a reliable measure of a patient’s true infection state. Laboratory testing is highly accurate, requires little human judgment, and has been established as the gold standard for diagnosis. However, test results come with a delay of about three days (Schmiemann et al., 2010). This test can confirm treatment decisions *ex post*, ensure full information is available to adjust treatment later, and provide antibiotic resistance information.

In primary care, no machine learning tools have so far been implemented for the treatment of UTI. An implementation could be feasible in telemedicine services, pharmacies, or primary care clinics in a health care system with interconnected IT systems across providers. In 2019, the UK National Health Service trialed a smartphone app where an antibiotic, nitrofurantoin or trimethoprim, could be obtained based on symptom reports and a dipstick result without seeing a physician.^4^ In the UK study, while symptoms and rapid test results were observed, no patient background data and no expert physician input could be used. Administered prescriptions could not be evaluated because the true sickness condition was not assessed. Hence, only the change in prescriptions was documented, lacking an evaluation of patients’ health outcomes.

## Danish administrative data and laboratory test results

### Danish national registries

The administrative data provided by Statistics Denmark cover all citizens and residents in Denmark between January 1st, 2002, and December 31st, 2012. The demographic data from the Danish Civil Registry (*Det Centrale Personregister, CPR*) includes gender, age, municipality, immigration status and place of origin, marriage, and family status. It provides a unique person identifier which facilitates accurate linkage of patients between the Danish national registers. It also includes household member identifiers which allow us to link the patient’s family and household members including their demographic and administrative data. We also obtain information on employment (*Integrerede Database for Arbejdsmarkedsforskning, IDA*) and education (*Uddannelseregister, UDDA*).

The prescription drug register (*Lægemiddeldatabasen, LMDB*) contains each individual’s complete purchase history of systemic antibiotics, including the date of purchase, patient and prescribing physician identifiers, and product information. The hospitalization data (*Landspatientregisteret, LPR*) comprise all patient contacts with hospitals, including ambulatory visits. The data include admission and discharge information, procedures performed, type of hospitalization (ambulatory, emergency, etc), diagnoses, and the number of bed days. The claims data (*Sygesikringsregisteret, SSR*) cover all medical services provided to the population of patients in primary care, including consultation week, services provided, and physician fees. Primary care providers are identified via unique clinic identifiers that can be linked to physicians’ personal identifiers (*Yderregister, YDER*).

### Microbiological laboratory data

Herlev Hospital and Hvidovre Hospital, two major hospitals in Denmark’s capital region covering a catchment area of roughly 1.7 million people, provide all test results from their clinical microbiological laboratories between January 1st, 2010, and December 31st, 2012. The data contain patient and clinic identifiers as well as information on test type, sample date, arrival date at the laboratory, result date, isolated bacteria, and antibiotic-specific resistances of isolated bacteria.

The laboratory test data are central because they reveal bacterial presence in a urine test sample, the outcome we aim to predict. According to the Danish guidelines urinalysis should only be performed in patients with signs and symptoms of UTI.^5^ The test procedure takes 3.1 days on average, during which physicians are uninformed about the result. Since we know the precise timing of test acquisitions, prescription purchases, and the test response date, we can determine physicians’ treatment decisions before being informed about test outcomes.

### Analysis sample

Overall, the data contain 2,579,617 biological samples submitted for testing in the capital region by both general practitioner clinics and hospitals. Urine samples constitute 477,609 samples out of which 156,694 are marked as general practitioners by the laboratory. Some clinics submit mainly specialist fee claims to the health care system. We drop these to ensure the sample includes only general practitioners. To focus on consultations that constitute a first contact with a physician, we exclude observations where a patient received a systemic antibiotic prescription or had a laboratory test conducted within 4 weeks before the observed test date. In these situations, physicians are unlikely to hold prior diagnostic information and must prescribe under uncertainty. By considering such initial consultations, we exclude potentially complicated treatment spells where patients are tested in later stages. We also avoid patients in long-term treatment, potentially due to severe antibiotic resistance problems. Additionally, we exclude urine samples collected during pregnancy as the vast majority of these are mandatory routine checks and do not represent UTI consultations. The final analysis sample consists of 65,919 initial consultations where a urine sample was sent to a laboratory for testing from 583 primary care clinics.

### Laboratory test outcomes and prescribing

We consider binary test outcomes that indicate whether bacteria are isolated in patients’ urine samples and do not focus on specific bacterial species. We observe when a test is acquired from the patient at an initial consultation and the initial prescription decision when a prescription for a systemic antibiotic is purchased at a pharmacy on the test day or the day after.^6^

Table 1 shows that the bacterial rate and prescription rate remain stable at 37-39 percent over the three sample years. This suggests that physicians match antibiotic prescriptions to bacterial infections very well at the initial consultation. Yet, the prescribing rates conditional on test outcome show that this is not the case. Physicians only prescribe antibiotics at initial consultations to 61 percent of patients with bacterial infections, implying underprescribing to 39 percent. Conversely, 26 percent of patients with a negative test result receive an antibiotic at the initial consultation, defined as overprescribing. Hence, the descriptives indicate a potential for improving physician decisions in treating UTI patients.

**Table 1:** Summary statistics for laboratory tests and initial antibiotic prescribing

	All tested			Positive test		Negative test	
	N	Bacterial rate	Prescribing rate	N	Prescribing rate	N	Prescribing rate
2010	17,513	0.37	0.39	6,411	0.60	11,102	0.27
2011	21,237	0.39	0.39	8,305	0.60	12,932	0.25
2012	27,169	0.39	0.39	10,510	0.61	16,659	0.25
Total	65,919	0.38	0.39	25,226	0.61	40,693	0.26

## Machine learning and physician decisions

### Predicting bacterial UTI using administrative data

We use the machine learning algorithm XGBoost (Hastie et al., 2009; Chen & Guestrin, 2016) to relate patient *i*’s covariates $x_i$ to the binary laboratory bacterial test outcome, $y_i$. XGBoost is an implementation of the extreme gradient boosted regression tree method that provides a non-parametric risk prediction. The vector $x_i$ contains 1,557 patient-specific covariates that may, in principle, be observable to the physician at the time of consultation.^7^ The covariates in the prediction model include each patient’s past medical outpatient claims, antibiotic purchases, microbiological test results, a rich set of characteristics such as gender, age, employment, education, income, civil status, and more, as well as the same information on each individuals’ household members. To account for clinic-specific practice styles, e.g. in sending test samples to the laboratory, we also include clinic identifiers, clinic-level past average resistance, and regional prescribing rates.

We use data from 2010 for hyperparameter tuning and create out-of-sample predictions for January 2011 to December 2012. Table 4 in Appendix A.1 reports the tuning results. To use the most recent historical data relative to a consultation, we retrain the XGBoost algorithm at a monthly frequency. Figure 4 in Appendix A.2 illustrates the data partitions used for hyperparameter search, training, and out-of-sample predictions. Table 5 in Appendix A.3 shows that sample sizes, bacterial and prescribing rates, risk predictions, and out-of-sample AUC are stable across partitions.

We report three measures of predictor importance for XGBoost – gain, frequency, and cover – in Fig. 5 and Table 6 in Appendix A.4. Across these measures, age, gender, clinic identifier, and recent antibiotic prescriptions are among the top 30 predictors reported in Table 6 in Appendix A.5. Further important predictors include a patient’s most recent antibiotic resistance results, clinic-specific resistance levels, regional prescription intensity, hospital stays, as well as a patient’s education, immigration status, and origin country. While many plausible narratives may relate these predictors to bacterial outcomes, machine learning algorithms do not have causal content and so we refrain from further interpretation.

The AUC is 0.721 for the risk predictions in the years 2011 and 2012 with the associated ROC curve reported in Fig. 6 in Appendix A.6. This AUC value falls in the ranges of prediction quality in the literature, for example Mullainathan and Obermeyer (2022) with 0.69 for heart attacks, Kleinberg et al. (2018a) with 0.707 for risk of recidivism, and between 0.56 and 0.83 for predicting antibiotic resistance conditional on the presence of bacteria in Yelin et al. (2019) and Kanjilal et al. (2020).

**Fig. 1:**
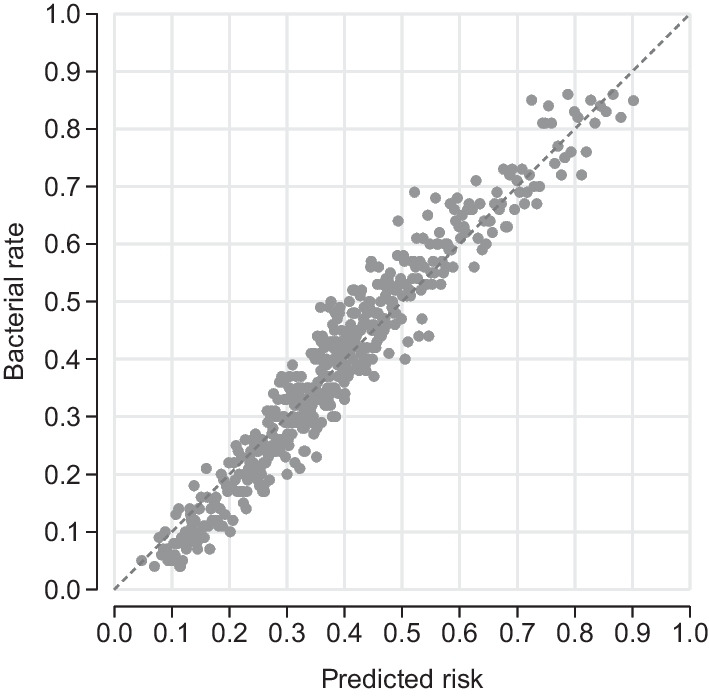
Laboratory test outcomes relative to predicted risk of bacterial UTI. Circles represent bins of 100 patients sorted by predicted risk

Figure 1 shows machine learning predicted risk, $m(x_i)$, and test outcomes for all out-of-sample test observations. We sort all patients by their predicted risk and compute average bacterial outcomes for consecutive bins of 100 patients. One bin is represented by one circle. Outcomes are close to the 45-degree line throughout the risk distribution, showing that the algorithm on average correctly predicts bacterial risk.

Our implementation is standard with the exception that we cannot split our data randomly into training and out-of-sample partitions using k-fold cross-validation. In practical applications, the prediction function must be constructed at or before the clinical consultation using historical data only. Splitting the data randomly could lead to spill-overs across time as past outcomes may be predicted using a model trained on future observations. To verify that our monthly updating of XGBoost does not result in overfitting, we also generate risk predictions for 2011 and 2012 training XGBoost exclusively on 2010 data. Even though we forego the use of increasing amounts of training data over time, this static approach results in an out-of-sample AUC of 0.709, only slightly below the value achieved using the main procedure.

A further potential source of overfitting may be that XGBoost recovers overly flexible conditional expectation functions on high-dimensional data. To insure against this risk of overfitting and inspect the relevance of model uncertainty, we reproduce our prediction exercise using parametric logistic LASSO. Using the same tuning and training procedure as described for XGBoost, we obtain an out-of-sample AUC of 0.707, which is just below the value achieved using XGBoost.^8^

Finally, while we cannot verify if the quality of machine learning predictions extrapolates beyond our sample, we can provide a partial assessment. Figure 7 (a) in Appendix A.7 shows the distribution of risk predictions for a subset of the general population sampled on a random day with no consultation.^9^ This distribution resembles the risk distribution in the analysis sample for patients without a bacterial infection. A notable difference is the larger density at low-risk predictions for the random population sample, which is driven by a larger proportion of men who on average exhibit lower risk of UTI. Analogously, Fig. 7 (b) in Appendix A.7 shows the distribution of risk predictions for patients who were prescribed a UTI-indicated antibiotic but are not in our analysis sample because no laboratory sample was collected.^10^ The distribution of risk predictions closely resembles the analysis sample for patients with a bacterial infection. These observations suggest that the prediction model may be informative for patients outside of the analysis sample.

### Bacterial rate conditional on predicted risk and physician prescribing

**Fig. 2:**
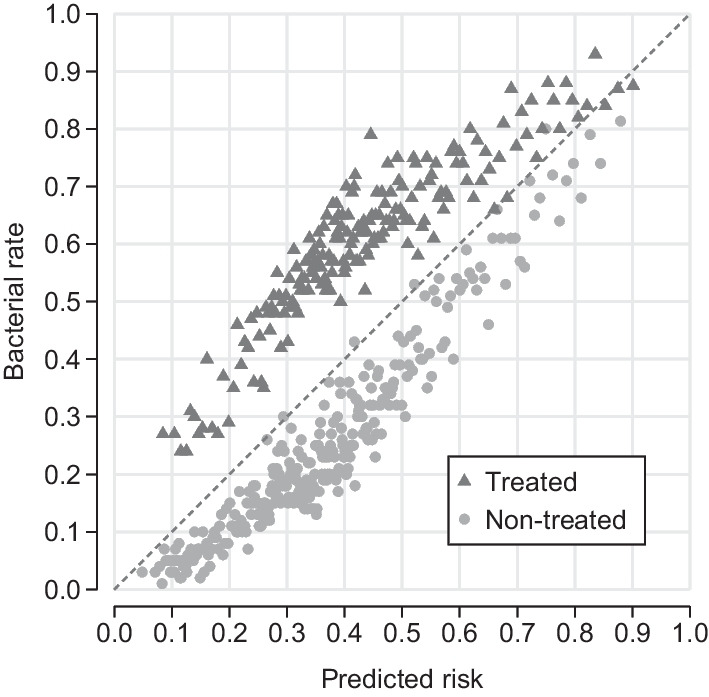
Laboratory test outcomes relative to predicted risk of bacterial UTI conditional on antibiotic prescribing prior to receiving test results. Circles and triangles represent bins of 100 patients sorted by predicted risk conditional on treatment

Motivated by the trade-off between the benefit and the social cost of antibiotic use, we focus on the binary choice of prescribing an antibiotic and not on molecule choice. Figure 2 splits the sample into patients who received a prescription (treated) and those who did not receive a prescription (non-treated) at the initial consultation. Again, each group is sorted by predicted risk and arranged into bins of 100 patients. Hence, the figure shows test outcomes versus risk predictions conditional on antibiotic prescribing prior to receiving test results. Conditional on predicted risk, patients with an initial prescription have higher bacterial rates than patients without an initial prescription. Hence, physicians appear to have diagnostic information that the machine learning algorithm does not capture. For example, point-of-care testing and symptom assessment provide instant, albeit imperfect, diagnostic information that is not included in administrative data. The difference in bacterial rates is largest for intermediate predicted risk, which represents the set of patients for which machine learning predictions are the least informative.

Even though physicians appear to have important private diagnostic information, prescriptions often do not match the true test outcomes. On average, 39.6 percent of patients who received an antibiotic did not have a bacterial infection and the overprescribing rate varies drastically with predicted risk. Among the 100 treated patients with the lowest predicted risk, the leftmost triangle in Fig. 2, only 27 patients had a bacterial infection resulting in 73 percent overprescribing. In contrast, 87.5 patients had a bacterial infection among the 100 treated patients with the highest predicted risk. Among the untreated, 25.1 percent of patients have bacterial infections. The error rate again varies with predicted risk showing an increasing bacterial rate for the non-treated patients as predicted risk increases. Among the 100 non-treated patients with the highest predicted risk, the rightmost circle on Fig. 2, 81 patients had a bacterial infection. These observations indicate that the match between prescriptions and bacterial infections can be improved at the extremes of the risk prediction range where machine learning classification accuracy is high and physician decisions reflect considerable over- and underprescribing.

## Designing policies to improve antibiotic prescribing

### Payoff from antibiotic prescribing

Our investigation centers on antibiotic prescription decisions for suspected UTI made during initial consultations in general practice clinics. Specifically, we focus on these first visits of sickness spells where urine samples were collected for laboratory testing. Test results enable the validation of initial treatment decisions as well as subsequent treatment that aligns with the patient’s initially unobserved sickness state. However, the delay in treatment during the waiting period, 3.1 days on average, incurs a substantial cost for patients with bacterial infections, prompting physicians to consider initiating antibiotic treatment at the initial consultation before receiving test results. Conversely, physicians often hesitate to prescribe antibiotics under uncertainty, as antibiotics only exhibit curative effects for bacterial infections, and as all antibiotic consumption advances antibiotic resistance regardless of the patient’s infection status. Thus, physicians grapple with a vital trade-off during the initial consultation: weighing the potential curative benefits of antibiotics against the cost of promoting antibiotic resistance (Adda, 2020). To formalize this trade-off, we define the realized payoff as a function of the prescription decision $d \in \{0,1\}$ during an initial consultation:

1 $$\begin{aligned} \pi (d; y) = - \alpha y(1-d) - \beta d , \end{aligned}$$

where $y \in \{0,1\}$ indicates whether the patient has a UTI. The parameter $\alpha >0$ is the relative weight on the patient’s sickness cost while awaiting the test result and the parameter $\beta >0$ reflects the relative resistance-promoting cost of prescribing.^11^

### Algorithm-supported prescription policies

We denote algorithm-supported counterfactual prescribing policy by $\delta _i$. For the set of patients $\mathcal {I}$, a policy can be evaluated using the aggregate payoff differences between the counterfactual prescription rule and the observed prescription choices:

2 $$\begin{aligned} \Pi = \sum _{i \in \mathcal {I}} \left[ \pi (\delta _i; y_i) -\pi (d_i; y_i) \right]&= \alpha \sum _{i \in \mathcal {I}} y_i \left( \delta _i-d_i \right) \; - \; \beta \sum _{i \in \mathcal {I}} (\delta _i - d_i) . \end{aligned}$$

The first term on the right-hand side of Eq. 2 is the change in the number of prescribed antibiotics to patients with a bacterial infection, while the second term is the change in overall antibiotic use. We aim to evaluate policies motivated by the broad public health objective of reducing antibiotic use. However, the policymaker’s preferred outcome depends on $\alpha$ and $\beta$ which are, in general, unknown. To make progress, we adopt an approach inspired by Kleinberg et al. (2018a) and focus on counterfactual prescribing that keeps the number of treated bacterial infections unchanged and minimizes overall antibiotic use.^12^ Observing Eq. 2, this approach guarantees an increase in payoffs for any $\alpha$ and $\beta$.

We introduce and evaluate two types of prescription policies. First, we explore full automation, that is a complete replacement of physician prescribing by an algorithm, entailing policies of the form:

3 $$\begin{aligned} \delta \left( m(x_i), k \right) \;\; = \;\; {\left\{ \begin{array}{ll} \;\; 0 &{} \text {if } m(x_i) \le k, \\ \;\; 1 &{} \text {if } k < m(x_i) . \end{array}\right. } \end{aligned}$$

Here, $m(x_i)$ is the machine learning risk prediction for patient *i* based on observables $x_i$, and *k* is a threshold parameter. The resulting prescription rules become step functions, where prescriptions are never given below the cut-off *k* and always given above. Appendix B.1 shows that these policies are optimal when ${{\,\textrm{E}\,}}(y)$ is increasing in the risk predictions. Inserting these rules into Eq. 2 combined with the aim to lower antibiotic use while maintaining the number of treated UTIs unchanged results in the following minimization problem:

4 $$\begin{aligned} \min _{k} \,\, \sum _{i \in \mathcal {I}} \delta _i(k) - d_i \quad \text {s.t.} \quad \sum _{i \in \mathcal {I}} y_i \left( \delta _i(k)-d_i \right) = 0. \end{aligned}$$

Full automation cannot make use of any diagnostic information expert physicians hold. Yet, Fig. 2 in Section 4.2 indicates that physician decisions hold important information conditional on machine learning-predicted risk. Hence, we investigate a second type of policy where the algorithm delegates a subset of decisions to physicians, implemented by prescription rules of the form:

5 $$\begin{aligned} \delta \left( m(x_i); k_L, k_H \right) \;\; = \;\; {\left\{ \begin{array}{ll} \;\; 0 &{} \text {if } m(x_i) \le k_L, \\ \;\; d_i &{} \text {if } k_L< m(x_i) < k_H, \\ \;\; 1 &{} \text {if } k_H \le m(x_i), \end{array}\right. } \end{aligned}$$

where $(k_L, k_H)$ are threshold parameters subject to $0 \le k_{L} \le k_{H} \le 1$. These rules postpone prescribing until test results are available for patients with low predicted risk, $m(x_i) \le k_L$, assign antibiotic prescriptions to patients with high predicted risk, $k_H \le m(x_i)$, and delegate decisions to physicians for intermediate risk, $k_L< m(x_i) < k_H$. Appendix B.2 shows that these policies are optimal when ${{\,\textrm{E}\,}}(y | d)$ is increasing in the risk predictions for both $d=1$ and $d=0$.^13^ Inserting these rules in Eq. 2 combined with the aim to lower antibiotic use while maintaining the number of treated UTIs unchanged results in the following minimization problem:

6 $$\begin{aligned} \min _{ k_L, k_H } \,\, \sum _{i \in \mathcal {I}} \delta _i(k_L, k_H) - d_i \quad \text {s.t.} \quad \sum _{i \in \mathcal {I}} y_i \left( \delta _i(k_L, k_H)-d_i \right) = 0. \end{aligned}$$

## Policy outcomes

We evaluate counterfactual policy outcomes relative to observed levels during the years 2011 and 2012. The 95 percent confidence intervals are derived by recomputing policy results across 1000 bootstrap samples while keeping patient risk predictions and policy parameters constant.

### Full automation based on machine learning predictions

Table 2 displays counterfactual policy outcomes in the absence of physician input. In this scenario, the optimal policy administers antibiotic prescriptions to all patients with a predicted risk equal to or higher than 0.405 and delays for those with a risk prediction below. This results in the reversal of 39.7 percent of observed physicians’ decisions. Notably, attempting to maintain a constant number of treated UTI cases hinders the reduction of antibiotic use when prescribing is done by the algorithm alone. Instead, counterfactual antibiotic usage experiences a 7.1 percent increase, accompanied by a 17.9 percent rise in overprescribing.^14^ These findings emphasize the need to integrate machine learning predictions with physician expertise for effective policy enhancements, even when high-dimensional individual-specific data is employed to generate patient-specific risk predictions.

**Table 2:** Counterfactual outcomes for 2011 and 2012, full automation

*k*	0.405
Change in treated UTI, in %	$0.0 \ \left[ \ -1.7, \ \ \ 1.8 \right]$
Change in antibiotic use, in %	$7.1 \ \left[ \ \ \ \ 5.6, \ \ \ 8.6 \right]$
Change in overprescribing, in %	$17.9 \ \left[ \ \ 15.1,\ 20.8 \right]$
Physician decisions overruled, in %	$39.7 \ \left[ \ \ 39.3,\ 40.2 \right]$

95% confidence intervals are based on 1000 bootstrap samples of 2011 and 2012 where machine learning predictions and the policy parameter *k* remain fixed

### Combining machine learning predictions and physician decisions

Table 3 displays outcomes achieved through the synergy of machine learning predictions and physician delegation. The optimal policy parameters, set to maximize the reduction in antibiotic use without changing the number of treated UTI patients, are $k_L=0.320$ and $k_H=0.601$. Accordingly, antibiotic treatment is delayed by the algorithm for patients with risk predictions below 0.320 and assigned by the algorithm when risk predictions exceed 0.601. Within the middle-risk range, prescription decisions are delegated to physicians. This approach results in an 8.1 percent reduction in overall antibiotic use and a 20.3 percent decrease in overprescribing relative to observed decisions. Physicians’ decisions are overruled and reversed in 15.0 percent of cases, with 52.8 percent of all consultations delegated to physicians.^15^

We provide three further sets of results that are informative for considering a potential implementation. First, even though we follow the literature in how we evaluate policy outcomes (Kleinberg et al., 2015, 2018a; Currie & MacLeod, 2017; Mullainathan & Obermeyer, 2022), parameters $k_L$ and $k_H$ would need to be fixed ahead of time in an actual implementation. In Appendix D, we show that it is feasible to obtain our results by sufficiently frequently updating policy parameters. Second, we focus on reducing antibiotic use without decreasing the number of treated UTI, but other objectives may be desirable. In Appendix E, we show outcomes for the complete set of objectives policy-makers may define concerning antibiotic use and the number of treated UTI. Finally, in Appendix F, we show how group fairness can be achieved by conditioning the policy objective on observable patient characteristics, but at the cost of some reductions in overall efficiency gains.^16^

**Table 3:** Counterfactual outcomes for 2011 and 2012, optimal delegation

$k_L$	0.320
$k_H$	0.601
Change in treated UTI, in %	$0.0 \ \left[ \ \ -1.0, \ \ \ \ \ 1.0 \right]$
Change in antibiotic use, in %	$-8.1 \ \left[ \ \ -8.9, \ \ -7.2 \right]$
Change in overprescribing, in %	$-20.3 \ \left[ -21.7, -18.8 \right]$
Physician decisions overruled, in %	$15.0 \ \left[ \ \ \ 14.7, \ \ \ 15.3 \right]$
Patients delegated to physicians, in %	$52.8 \ \left[ \ \ \ 52.3, \ \ \ 53.3 \right]$
Consultations	48, 406
UTIs	18, 815
Treated UTIs	11, 402
Antibiotic prescriptions	18, 872
Overprescribing	7, 470

95% confidence intervals are based on 1000 bootstrap samples of 2011 and 2012 where machine learning predictions and the policy parameter $(k_{L},k_{H})$ remain fixed

### Physician private diagnostic information

The complementarity between physician decisions and machine learning risk predictions is apparent from the superior performance of the policy with optimal delegation over full automation. In this section, we take a closer look at physician private information as a potential factor contributing to physician performance. We focus on one main source of private information: in-house diagnostic testing in the form of rapid point-of-care dipstick tests and microscopy analysis. Outcomes of in-house diagnostic tests conducted during these consultations are typically not encoded in administrative data, preventing their use in training algorithmic risk predictions. However, we observe the utilization of dipstick and microscopy diagnostics during consultations which we can relate to a measure of physicians’ private information.

We define private diagnostic information as the difference between machine learning prediction errors, $\vert y_i-m(x_i) \vert$, and physician prescription errors, $\vert y_i-d_i \vert$, which yields

7 $$\begin{aligned} \iota _i = \vert y_i-m(x_i) \vert - \vert y_i-d_i \vert = ( d_i-m(x_i) ) y_i + (m(x_i)-d_i) (1-y_i). \end{aligned}$$

**Fig. 3:**
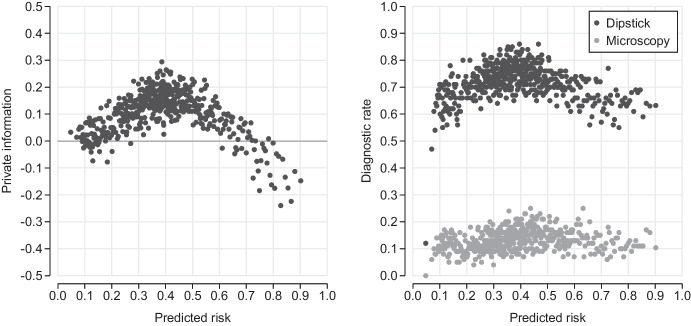
Physician private information relative to machine learning predictions (left) and the dipstick and microscopy diagnostic rate (right) as a function of predicted risk

**Fig. 4:**
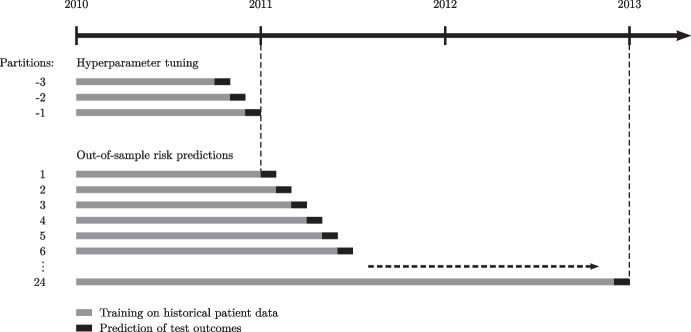
Outline of the data partitions used for hyperparameter tuning as well as the month-by-month progressing training and out-of-sample prediction partitions

**Fig. 5:**
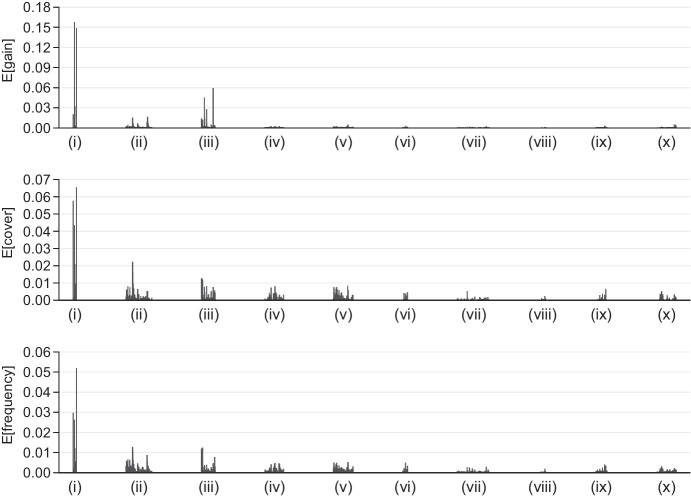
Average gain, cover and frequency over the 24 monthly XGBoost models

**Fig. 6:**
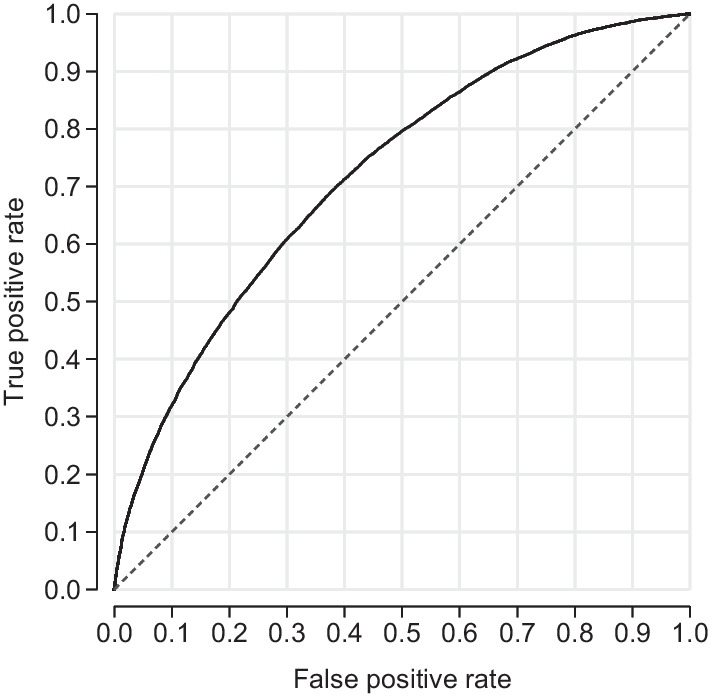
Receiver operating characteristic (ROC) curve for XGBoost. The ROC plots all trade-offs between true positive and false positive rates achievable by a prediction technology for a binary outcome. A technology with perfect predictions achieves a true positive rate of one and a false positive rate of zero. The dashed diagonal represents the ROC curve of a prediction technology which is as good as random draws, i.e. providing no information

**Fig. 7:**
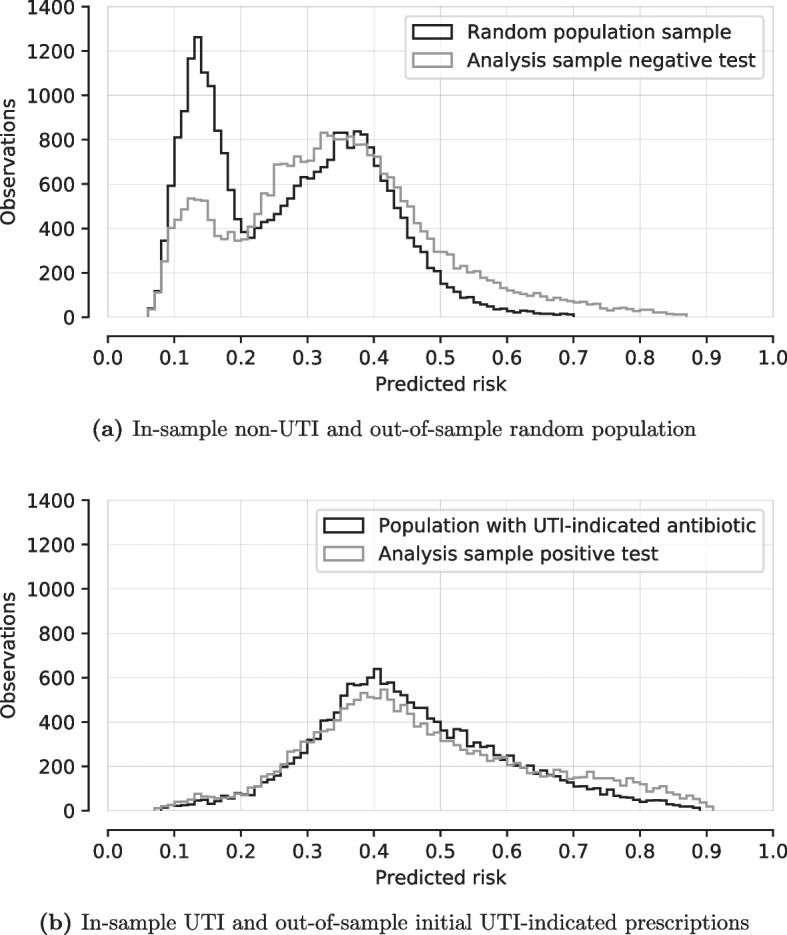
In-sample and out-of-sample predicted risk distributions. Bars with fewer than 10 patients have been removed due to anonymity restrictions. The samples without laboratory tests are drawn such that they have the same number of observations as the corresponding analysis sample for $y=0$ and $y=1$

This measure represents physicians’ diagnostic information relative to information recovered by machine learning predictions. The left panel of Fig. 3 shows the distribution of private diagnostic information $\iota _i$ for bins of 100 patients sorted on predicted risk. In line with our discussion of over- and under-prescribing, private information follows an inverted U-shape with low information in the low- and high-risk range but high private information in the intermediate-risk range.

The right panel of Fig. 3 shows the dipstick and microscopy rate across the risk range. On average, a dipstick diagnostic is used in 72 percent and microscopy in 13 percent of all consultations. Physicians perform more diagnostic tests at medium levels of predicted risk, where physicians have lower decision error rates compared to algorithmic decisions. This observation suggests that diagnostic tests at the point of care are an important source of private diagnostic information.

In typical health care settings, expert decision-makers hold context-specific private information beyond the reach of machine learning, as measured by $\iota _i$. In practice, multiple factors complicate the use of such information, including privacy concerns, legal considerations, a lack of standardization in diverse provider IT systems, inconsistencies in reporting, and simply an absence of (symptom) documentation. Implementing algorithmic decision rules that allow for delegation to human decision-makers, where they hold important private information, provides a way to overcome this challenge.

### Robustness to sample selection

Our analysis sample is selected in the sense that initial consultations are only included if a laboratory test was made. Suppose physicians only send urine samples in for laboratory testing when they have systematically high (low) private information. In that case, our results may represent only an upper (lower) bound of what an algorithm with delegation would achieve in the general population of initial UTI consultations. We assess the robustness of our results to test selection using two approaches.

**Fig. 11:**
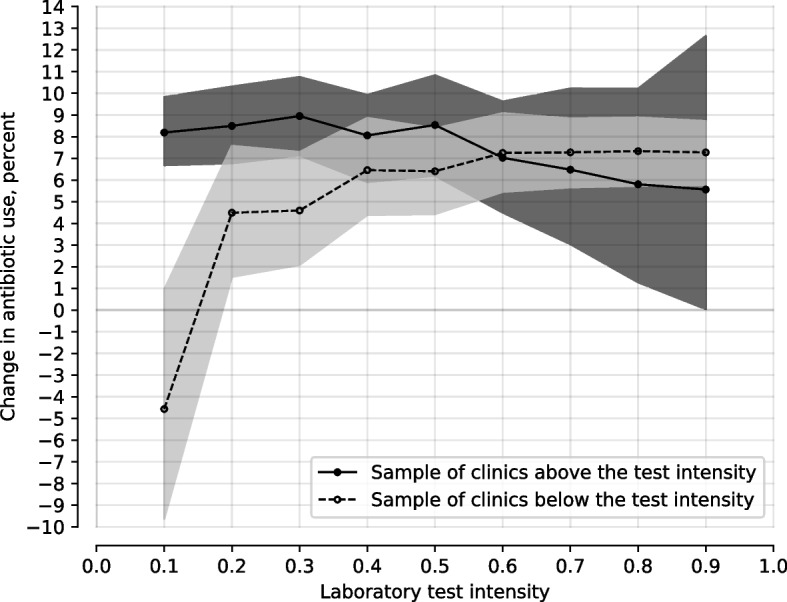
Policy outcomes by laboratory testing intensity, full automation

**Fig. 12:**
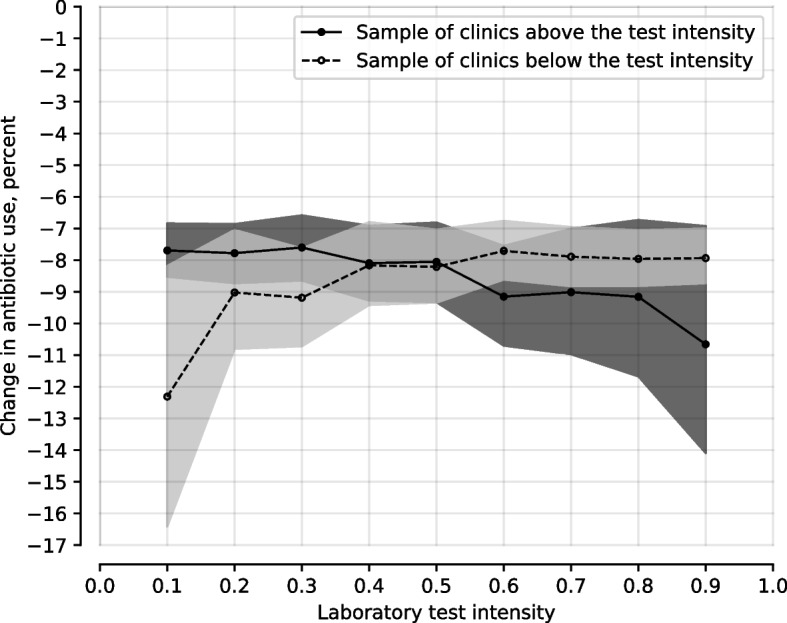
Policy outcomes by laboratory testing intensity, optimal delegation

**Fig. 13:**
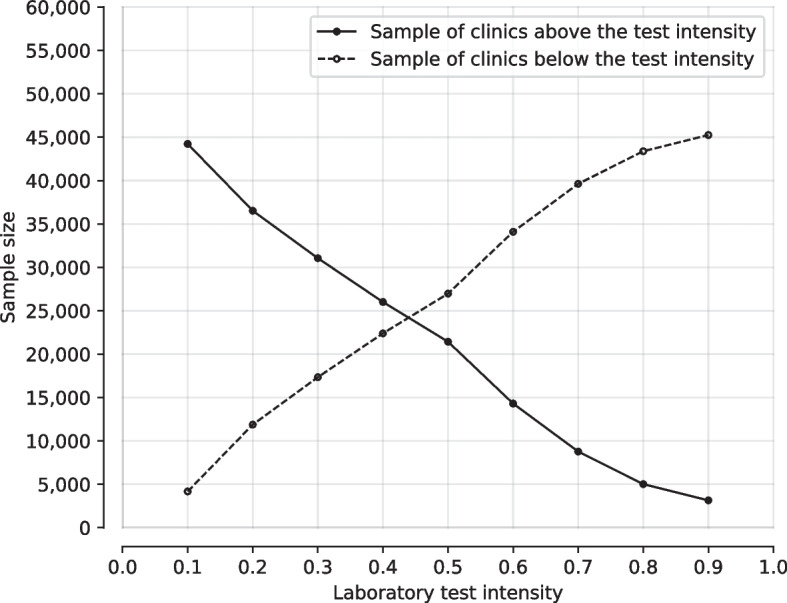
Sizes of samples split by laboratory test intensity. For a given test intensity, the two samples sum to the full sample size of 48,406

In the first, we make use of the cross-clinic variation in the propensity to use a laboratory test. We measure test intensity for each clinic by dividing the clinic’s number of laboratory tests by its number of initial UTI consultations.^17^ Figures 11 and 12 in Appendix H show the counterfactual reduction in antibiotic use conditional on varying test intensities for both types of policies. Figure 13 in Appendix H the associated sample sizes. The solid line shows results for all samples from clinics above or equal to the testing intensity threshold. The dashed line shows results for all samples from clinics below the threshold. Across sub-samples of clinics with varying test intensities, the policy results are close to our main results and their confidence intervals largely overlap.

In the second robustness check, we evaluate the algorithmic prescription policy on the random sample of the healthy general population as well as on the population of patients with UTI-indicated prescriptions without laboratory testing described in Section 4.1.

In the general population, one percent of all hypothetical consultations have a predicted risk above $k_H=0.601$ and, hence, would include an antibiotic prescription in the counterfactual policy. This false positive rate is significantly smaller than the share of antibiotics given for non-UTI cases in our main sample, where 5.6 percent of consultations have a predicted risk above $k_H$. Conversely, among patients with UTI-indicated prescriptions without laboratory testing, 15.2 percent have a predicted risk below $k_L=0.320$. The policy would delay antibiotic prescribing for these patients. This hypothetical false negative rate of 15.2 percent is comparable to the share of 17.7 percent of patients with a bacterial infection in our main sample with predicted risk below $k_L$.

These results suggest that potential sample selection is a limited threat to our results.

## Conclusion

The quality of prediction algorithms and available data is improving at a rapid pace. In this paper, we document the complementary role of machine learning methods for decision-making in a typical context of primary health care provision. We show that decision rules based on machine learning predictions using administrative data may provide a path to improve antibiotic prescribing. Antibiotic prescribing has important societal implications due to increasing antibiotic resistance driven by inefficient antibiotic use. While counterfactual policies based on machine learning predictions alone do not deliver improvements, antibiotic use can be reduced by delegating decisions between physicians and machine learning where each is most certain. Systems should therefore be designed with the decision-improving input human experts can provide in mind.

We consider the specific case of UTI in primary care in Denmark, a country with a record of low antibiotic use (Goossens et al., 2005). Relating the potential reductions in prescribing to the national action plan initiated by the Danish government in 2017, which aimed to reduce overall antibiotic prescribing by one-third within three years (Danish Ministry of Health, 2017), the reduction of 8.1 percent would achieve one-fourth of this goal. While our analysis may be challenging to implement in other countries due to the lack of linked data, we suspect the potential reductions we find present a lower bound of what may be achievable in other institutional settings. One limitation is that we consider only initial consultations in which a laboratory test was used. This restriction enables us to observe the ground truth irrespective of physicians’ initial treatment decisions, allowing us to evaluate physicians’ decisions. We provide evidence that our results may not be limited to this specific sample but further research is needed on new data from varying contexts.

While we focus on human-AI complementarity for decision outcomes, the considered policy may also help increase productivity. Because a share of decisions does not require human input, physicians and patients may save time and effort. These valuable resources may instead be used on more productive physician-patient interactions and other diagnostic tools at the point of care.

One promising avenue for further research is the analysis of experts’ behavioral reactions to prediction-based policies. For example, physicians’ incentives to exert effort in gathering information are likely to change or they may attempt to conform to the decisions made by the algorithm. Such potential strategic reactions by human decision-makers can affect policy outcomes and call for careful evaluation of interventions in the field.

## Figures and Tables

**Fig. 8:**
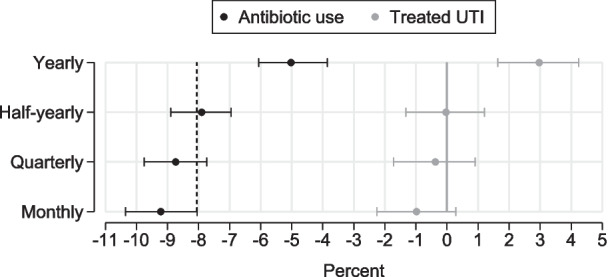
Policy results for 2012, updating parameters *ex ante* at yearly, half-yearly, quarterly and monthly intervals. The dashed lines show the main results in Table 3

**Fig. 9:**
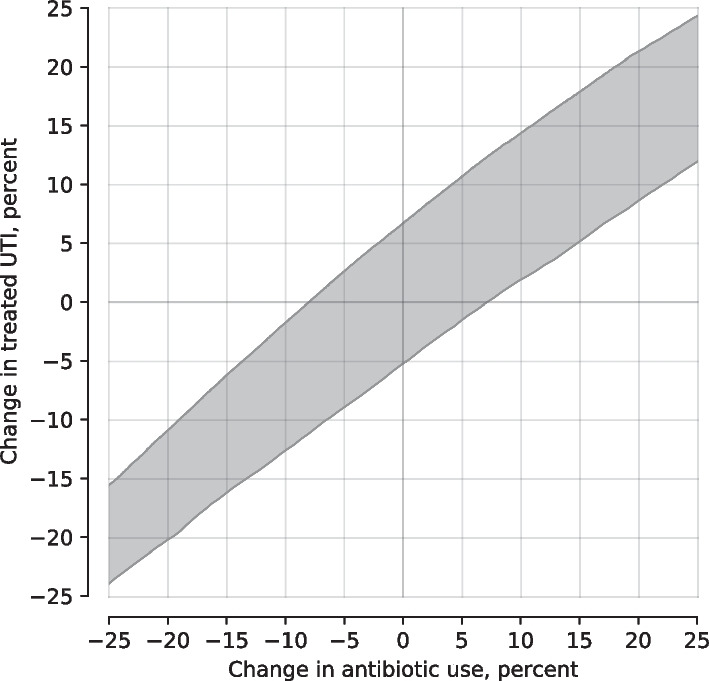
Policy outcomes as a function of policy parameters $(k_L,k_H)$

**Fig. 10:**
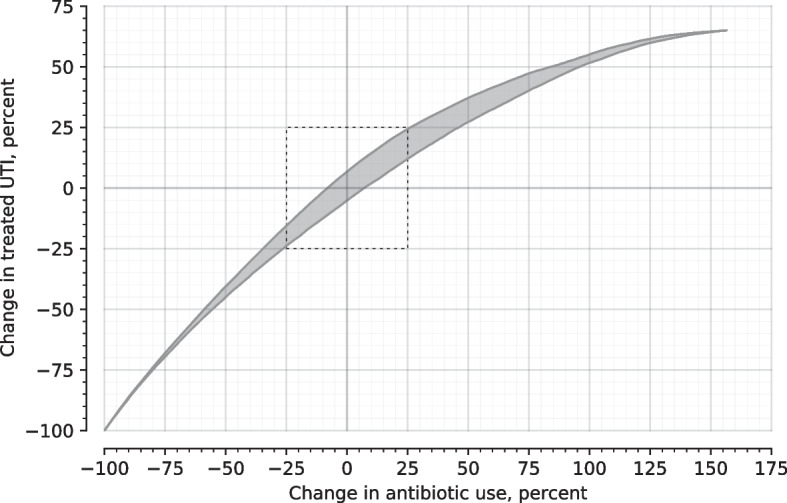
The set of all policy outcomes as a function of the policy parameters $(k_L,k_H)$ for 2011 and 2012. The dashed rectangle shows the policy outcomes highlighted in Figure 9 in the main text

## Appendix A Machine learning

### A.1 Hyperparameters

**Table 4:** Top 5 hyperparameter search results

Rank	Rounds	Learning rate	Tree depth	Avg. AUC
1	446	0.04	3	0.69997
2	353	0.05	3	0.69956
3	604	0.02	4	0.69949
4	434	0.04	4	0.69932
5	739	0.03	3	0.69913

We restrict the hyperparameter search space to the learning rate, the number of boosting rounds and the tree depth. The AUC is averaged over the three hyperparameter partitions

### A.3 Data partitions

**Table 5:** Summary statistics for data partitions

	Training					Prediction						
Partition	N	E[*y*]	E[*d*]	E[$d {\mid } y{=}1$]	E[$d {\mid } y{=}0$]	N	E[m(x)]	E[*y*]	E[*d*]	E[$d {\mid } y{=}1$]	E[$d {\mid } y{=}0$]	AUC
-3	12,867	0.37	0.39	0.61	0.27	1,618		0.37	0.39	0.58	0.27	
-2	14,485	0.37	0.39	0.60	0.27	1,705		0.37	0.38	0.60	0.26	
-1	16,190	0.37	0.39	0.60	0.27	1,323		0.36	0.42	0.62	0.30	
1	17,513	0.37	0.39	0.60	0.27	1,755	0.36	0.36	0.37	0.58	0.25	0.71
2	19,268	0.37	0.39	0.60	0.27	1,510	0.37	0.37	0.38	0.59	0.26	0.73
3	20,778	0.37	0.39	0.60	0.27	1,811	0.37	0.38	0.37	0.57	0.25	0.71
4	22,589	0.37	0.39	0.60	0.26	1,413	0.37	0.40	0.40	0.60	0.27	0.70
5	24,002	0.37	0.39	0.60	0.26	1,864	0.38	0.40	0.37	0.55	0.24	0.71
6	25,866	0.37	0.39	0.60	0.26	1,753	0.40	0.41	0.38	0.58	0.24	0.73
7	27,619	0.37	0.39	0.59	0.26	1,257	0.41	0.41	0.45	0.68	0.29	0.69
8	28,876	0.37	0.39	0.60	0.26	1,936	0.40	0.40	0.38	0.61	0.23	0.70
9	30,812	0.38	0.39	0.60	0.26	2,092	0.39	0.39	0.40	0.62	0.26	0.72
10	32,904	0.38	0.39	0.60	0.26	2,027	0.39	0.39	0.40	0.61	0.26	0.70
11	34,931	0.38	0.39	0.60	0.26	2,166	0.39	0.39	0.37	0.58	0.24	0.71
12	37,097	0.38	0.39	0.60	0.26	1,653	0.39	0.41	0.40	0.61	0.25	0.72
13	38,750	0.38	0.39	0.60	0.26	2,244	0.40	0.39	0.39	0.61	0.24	0.74
14	40,994	0.38	0.39	0.60	0.26	1,914	0.40	0.38	0.37	0.62	0.23	0.72
15	42,908	0.38	0.39	0.60	0.26	2,202	0.39	0.36	0.36	0.59	0.24	0.71
16	45,110	0.38	0.39	0.60	0.26	1,683	0.40	0.40	0.41	0.63	0.25	0.73
17	46,793	0.38	0.39	0.60	0.26	2,064	0.40	0.37	0.38	0.60	0.25	0.74
18	48,857	0.38	0.39	0.60	0.26	2,410	0.39	0.38	0.38	0.59	0.26	0.73
19	51,267	0.38	0.39	0.60	0.26	1,645	0.41	0.43	0.44	0.65	0.28	0.72
20	52,912	0.38	0.39	0.60	0.26	2,759	0.40	0.40	0.41	0.62	0.27	0.72
21	55,671	0.38	0.39	0.61	0.26	2,506	0.38	0.40	0.39	0.60	0.25	0.73
22	58,177	0.38	0.39	0.61	0.26	2,770	0.39	0.39	0.40	0.62	0.27	0.72
23	60,947	0.38	0.39	0.61	0.26	3,018	0.39	0.37	0.37	0.60	0.24	0.74
24	63,965	0.38	0.39	0.61	0.26	1,954	0.39	0.39	0.41	0.62	0.27	0.73

### A.4 Predictor importance

Gain, cover, and frequency provide measures of predictor importance (Chen et al., 2022). Variables in Fig. 5 are listed by groups based on their administrative data sources:

(i) patient demographics, test timing and assigned physician identifier
(ii) patient prescriptions and assigned physician’s average antibiotic use
(iii) patient laboratory tests and assigned physician’s average test results
(iv) patient hospitalizations
(v) patient primary care claims
(vi) Household characteristics
(vii) Household member prescriptions
(viii) Household member laboratory tests
(ix) Household member hospitalizations
(x) Household member primary care claims

### A.5 Top 30 predictors by gain, cover, and frequency

**Table 6:** Top 30 predictors by gain, cover, and frequency

	Sorted by gain			Sorted by cover			Sorted by frequency		
	Predictor	Group	E[gain]	Predictor	Group	E[cover]	Predictor	Group	E[frequency]
1	Gender	(i)	0.1572	Age	(i)	0.0652	Age	(i)	0.0518
2	Age	(i)	0.1482	Clinic identifier	(i)	0.0574	Clinic identifier	(i)	0.0296
3	Resistance to J01XE01 (1)	(iii)	0.0588	Gender	(i)	0.0433	Gender	(i)	0.0261
4	Resistance to J01CA11 (1)	(iii)	0.0446	Prescription ATC code (1)	(ii)	0.0221	Prescription ATC code (1)	(ii)	0.0125
5	Immigration status	(i)	0.0321	Immigration status	(i)	0.0208	GP 6 months mean resistance	(iii)	0.0123
6	Resistance to J01DD13 (1)	(iii)	0.0274	Prescription ATC code (3)	(ii)	0.0155	Immigration status	(i)	0.0118
7	Clinic identifier	(i)	0.0197	Prescription ATC code (2)	(ii)	0.0146	GP all previous mean resistance	(iii)	0.0118
8	Days since prescription (4)	(ii)	0.0159	GP all previous mean resistance	(iii)	0.0125	Prescription ATC code (3)	(ii)	0.0107
9	Prescription ATC code (1)	(ii)	0.0147	GP 1 year mean resistance	(iii)	0.0122	GP 1 year mean resistance	(iii)	0.0086
10	Resistance to J01CA11 (2)	(iii)	0.0138	GP 6 months mean resistance	(iii)	0.0116	Days since prescription (1)	(ii)	0.0086
11	GP all previous mean resistance	(iii)	0.0133	Origin country	(i)	0.0096	Prescription ATC code (2)	(ii)	0.0083
12	GP 6 months mean resistance	(iii)	0.0115	Prescription ATC code (4)	(ii)	0.0095	Days since lab test (1)	(iii)	0.0075
13	Days since prescription (3)	(ii)	0.0104	Education	(i)	0.0092	Days since lab test (1)	(iii)	0.0068
14	GP 1 year mean resistance	(iii)	0.0091	Weeks since specialist (28)	(iv)	0.0082	Municipal DID of J01CF01	(ii)	0.0065
15	Days since prescription (2)	(ii)	0.0090	Resistance to J01DD13 (1)	(iii)	0.0081	Municipal DID of J01FA01	(ii)	0.0062
16	Prescription ATC code (2)	(ii)	0.0088	Hospital bed days (7)	(iv)	0.0079	Prescription ATC code (4)	(ii)	0.0061
17	Days since prescription (1)	(ii)	0.0076	Municipal DID of J01CF01	(ii)	0.0079	Municipal DID of J01EB02	(ii)	0.0058
18	Resistance to J01DD13 (2)	(iii)	0.0071	Resistance to J01CA11 (1)	(iii)	0.0076	Municipal DID of J01AA07	(ii)	0.0057
19	Prescription ATC code (3)	(ii)	0.0065	Claim of non-GP specialist (21)	(v)	0.0075	Education	(i)	0.0056
20	Prescription indication (2)	(ii)	0.0063	Resistance to J01XE01 (1)	(iii)	0.0074	Days since prescription (3)	(ii)	0.0054
21	Days since prescription (7)	(ii)	0.0063	Municipal DID of J01FA01	(ii)	0.0072	Origin country	(i)	0.0054
22	Prescription ATC code (4)	(ii)	0.0057	Claim of non-GP specialist (4)	(v)	0.0072	Weeks since specialist (28)	(v)	0.0050
23	Resistance to J01XE01 (2)	(iii)	0.0055	Hospital diagnose (9)	(iv)	0.0072	Claim of non-GP specialist (4)	(v)	0.0048
24	Prescription indication (3)	(ii)	0.0055	Employment industry	(i)	0.0071	Mother’s age	(vi)	0.0048
25	Prescription indication (4)	(ii)	0.0052	Prescription ATC code (8)	(ii)	0.0067	Weeks since specialist (30)	(v)	0.0048
26	Resistance to J01MA02 (1)	(iii)	0.0046	Prescription ATC code (7)	(ii)	0.0066	Claim of non-GP specialist (21)	(v)	0.0047
27	Weeks since GP visit, family (8)	(x)	0.0045	Days since hospital, family (8)	(ix)	0.0065	Hospital bed days (7)	(iv)	0.0046
28	Weeks since GP visit, family (17)	(x)	0.0045	Claim of non-GP specialist (17)	(v)	0.0064	Prescription indication (2)	(ii)	0.0044
29	Weeks since specialist (30)	(v)	0.0044	Prescription indication (3)	(ii)	0.0063	Resistance to J01XE01 (1)	(iii)	0.0044
30	Municipal DID of J01CF01	(ii)	0.0039	Claim of non-GP specialist (24)	(v)	0.0061	Days since hospital (1)	(iv)	0.0044

All variables are measured relative to the laboratory test date and refer to the patient unless otherwise specified by family relation, region, or clinic. Numbers in brackets indicate the recency of the observation. For instance, “prescription ATC code (3)” contains the ATC code (The Anatomical Therapeutic Chemical) of the patient’s 3rd most recent prescription relative to the test date. DID stands for defined daily dose per 1000 inhabitants per day and codes of the form J01**** are the ATC code of a specific antibiotic

## Appendix B Optimal policies

### B.1 Single-cutoff rule without delegation to physicians

We motivate the policy without delegation to physicians by observing the positive relation between *E*[*y*] and *m*(*x*) in Fig. 1. Assume the expected bacterial rate conditional on predicted risk is a strictly increasing and continuous function of predicted risk, i.e. $E \{ y \vert m(x) \} = f(m(x))$ with $f(0)=0$ and $f(1)=1$. Then, the optimal prescription policy, $\delta (m(x),k)$, relative to the relevant payoff parameters with $\frac{\beta }{\alpha } \in [0, 1]$ will satisfy

8 $$\begin{aligned} {\begin{matrix} \delta (m(x),k) = 1 &{} \quad \Leftrightarrow \quad {{\,\textrm{E}\,}}\{ \pi (\delta =1; y) \mid m(x) \}> {{\,\textrm{E}\,}}\{ \pi (\delta =0; y) \mid m(x) \} \\ &{} \quad \Leftrightarrow \quad {{\,\textrm{E}\,}}\{ y \mid m(x) \}> \frac{\beta }{\alpha } \\ &{}\quad \Leftrightarrow \quad m(x) > f^{-1} \left( \frac{\beta }{\alpha } \right) \equiv k . \end{matrix}} \end{aligned}$$

Since the inverse of a strictly increasing function on a bounded interval has an inverse that is also strictly increasing, the optimal policy is a step-function where larger values of $\frac{\beta }{\alpha }$ results in larger cut-off *k* and prioritizes reduction in antibiotic use at the cost of fewer treated UTI, and smaller values of $\frac{\beta }{\alpha }$ results in smaller cut-off *k* prioritizing more treated UTI at the cost of increasing antibiotic use.

### B.2 Two-cutoff rule with delegation to physicians

We motivate the form of our combined physician and algorithmic policy by observing Fig. 2 which shows $E \{ y \vert m(x) \}$ conditional on the physician’s decision. Assume $E \{ y \vert m(x), d=1 \} = g(m(x))$ is strictly increasing and continuous with $g(0)=0$ and $g(1)=1$. Similarly, assume $E \{ y \vert m(x), d=0 \} = h(m(x))$ is strictly increasing and continuous with $h(0)=0$ and $h(1)=1$. Lastly, assume $g(m(x))>h(m(x))$ for all $m(x) \in (0,1)$. In this case, the optimal prescription policy, $\delta (m(x),k_L,k_H)$, relative to the payoff parameters with $\frac{\beta }{\alpha } \in (0, 1)$ will overwrite physician decisions when

9 $$\begin{aligned} {\begin{matrix} \delta = 0 \mid d=1\! &{} \quad \Leftrightarrow \! \quad E \{ \pi (\delta =1; y) \mid m(x), d=1 \} \!\le \! E \{ \pi (\delta =0; y) \mid m(x), d=1 \} \\ &{} \quad \Leftrightarrow \! \quad E \{ y \mid m(x), d=1 \} \le \frac{\beta }{\alpha } \\ &{}\quad \Leftrightarrow \! \quad m(x) \le g^{-1} \left( \frac{\beta }{\alpha } \right) \equiv k_L, \end{matrix}} \end{aligned}$$

as well as when

10 $$\begin{aligned} {\begin{matrix} \delta = 1 \mid d=0\! &{} \quad \Leftrightarrow \! \quad E \{ \pi (\delta =1; y) \mid m(x), d=0 \} \!>\! E \{ \pi (\delta =0; y) \mid m(x), d=0 \} \\ &{} \quad \Leftrightarrow \! \quad E \{ y \mid m(x), d=0 \}> \frac{\beta }{\alpha } \\ &{}\quad \Leftrightarrow \! \quad m(x) > h^{-1} \left( \frac{\beta }{\alpha } \right) \equiv k_H . \end{matrix}} \end{aligned}$$

We used that the inverse of a strictly increasing function on a bounded interval has an inverse that is also strictly increasing. Since $g(k_L) = \frac{\beta }{\alpha } = h(k_H) < g(k_H)$, we must have that $k_L < k_H$ and that the optimal policy has an interval $(k_L, k_H)$ wherein it is optimal that the algorithm does not interfere with physician decisions.

## Appendix C Policy outcomes using LASSO for prediction

**Table 7:** Counterfactual outcomes for 2011 and 2012 using Lasso, full automation

*k*	0.389
Change in treated UTI, in %	$0.0 \ \left[ -1.7, \ \ \ 1.6 \right]$
Change in antibiotic use, in %	$11.3 \ \left[ \ \ \ 9.7, \ 12.9 \right]$
Change in overprescribing, in %	$28.7 \ \left[ \ 25.6, \ 32.0 \right]$
Physician decisions overruled, in %	$41.2 \ \left[ \ 40.8, \ 41.7 \right]$

95% confidence intervals are based on 1000 bootstrap samples of 2011 and 2012 where machine learning predictions and the policy parameter *k* remain fixed

**Table 8:** Counterfactual policy outcomes for 2011 and 2012 using Lasso, optimal delegation

$k_L$	0.300
$k_H$	0.633
Change in treated UTI, in %	$0.0 \ \left[ \ \ -0.9, \ \ \ \ \ 1.0 \right]$
Change in antibiotic use, in %	$-7.0 \ \left[ \ \ -7.7, \ \ -6.2 \right]$
Change in overprescribing, in %	$-17.6 \ \left[ -18.9, -16.3 \right]$
Physician decisions overruled, in %	$11.4 \ \left[ \ \ \ 11.1, \ \ \ 11.7 \right]$
Patients delegated to physicians, in %	$62.3 \ \left[ \ \ \ 61.9, \ \ \ 62.8 \right]$
Consultations	48, 406
UTIs	18, 815
Treated UTIs	11, 402
Antibiotic prescriptions	18, 872
Overprescribing	7, 470

95% confidence intervals are based on 1000 bootstrap samples of 2011 and 2012 where Lasso predictions and the policy parameter $(k_{L},k_{H})$ remain fixed

## Appendix D *Ex ante* policy parameters

For the main results in Table 3, policy parameters $k_L$ and $k_H$ are optimized *ex post*, following the literature (Bayati et al., 2014; Kleinberg et al., 2018a; Yelin et al., 2019; Hastings et al., 2020; Mullainathan & Obermeyer, 2022).^18^ That is, we solve Eqs. 4 and 6 after observing machine learning predictions, prescription choices, and test outcomes for 2011 and 2012. In a real-world application, both policy parameters would need to be determined ahead of time. There are many potential ways to go about this task, see for example Hazan (2022). Here, we show that simple ways to determine and update the policy parameters *ex ante* suffice to realize the policy results.

Specifically, we split the years 2011 and 2012 into intervals and set $k_L$ and $k_H$ *ex ante* for an interval using only data from the preceding interval. We implement this procedure for intervals of one year, that is using 2011 to determine policy parameters for 2012, as well as for intervals of one half-year, one quarter, and one month. As the first interval is set apart to fix policy parameters *ex ante*, the longest overlapping evaluation period needed for a comparision between the four interval definitions is the full year 2012. Figure 8 shows the *ex ante* counterfactual results with the added dashed vertical lines showing our main results from Table 3.

Yearly policy parameters cannot reproduce the main results. The number of correctly treated bacterial UTIs increases while the reduction in antibiotic use is substantially lower than in the main results. Yet, by updating policy parameters at half-yearly, quarterly, and monthly intervals, the main policy results, where parameters are set ex post, can be attained. Table 9 shows all *ex ante* and *ex post* 2012 policy results for each interval definition confirming that the different intervals do not result in significantly varying policy outcomes except for the yearly interval length.

**Table 9:** Policy results for 2012 with policy parameters set *ex ante* and *ex post*

	Change in antibiotic use (%)		Change in treated UTI (%)	
$k_L$, $k_H$ computed	*Ex post*	*Ex ante*	*Ex post*	*Ex ante*
Yearly	-8.6 [-9.8, -7.4]	-5.0 [-6.1, -3.9]	0.0 [-1.5, 1.4]	3.0 [1.6, 4.4]
Half-yearly	-8.7 [-9.7, -7.5]	-7.9 [-8.9, -6.9]	0.0 [-1.3, 1.3]	-0.0 [-1.4, 1.3]
Quarterly	-8.8 [-9.9, -7.6]	-8.7 [-9.9, -7.7]	0.0 [-1.3, 1.4]	-0.4 [-1.8, 1.0]
Monthly	-9.2 [-10.3, -8.1]	-9.3 [-10.3, -8.2]	0.0 [-1.4, 1.3]	-1.0 [-2.4, 0.3]

95% confidence intervals are based on 1000 bootstrap samples where machine learning predictions and policy parameters $(k_{L},k_{H})$ remain fixed

## Appendix E Alternative policy objectives

Motivated by common public health policy considerations, we have focused on the policy objective of reducing antibiotic use without treating fewer patients with bacterial UTI (WHO, 2012, 2014). Alternative policy objectives can be attained. Figure 9 shows the set of attainable changes in antibiotic use and the number of treated bacterial UTIs for all possible policy parameters $0 \le k_L \le k_H \le 1$. The full range can be seen in Fig. 10. The upper bound of this set represents the payoff-maximizing trade-offs between antibiotic use and treated UTIs.

In the upper left quadrant, antibiotic use is reduced while the number of treated bacterial UTIs is increased. In this region, any policymaker will prefer the counterfactual policy outcomes relative to the status quo regardless of policymaker preferences $\alpha >0$ and $\beta >0$. Our main result lies at the boundary of this region where the upper bound intercepts the horizontal axis. Here, the change in the number of treated bacterial infections is zero and the change in antibiotic use is $-8.1$ percent. Where the upper bound intersects the vertical axis, the counterfactual policy keeps the number of antibiotic prescriptions at initial consultations constant but increases the number of treated bacterial infections by 7.0 percent. Although the overall use of antibiotics is unchanged, the more efficient use of antibiotics still leads to a reduction in overprescribing by 10.5 percent.

Larger reductions in antibiotic use can be obtained, but not without decreasing the number of treated bacterial infections. For instance, reducing antibiotic use by 20.0 percent would require 10.8 percent of patients with bacterial infections, who were given antibiotics, to delay treatment until test results are available. Analogously, a 20 percent increase in treated UTI could be attained but only with a 17.9 percent increase in antibiotic use. Ultimately, policymaker preferences unobserved to us determine the optimal trade-off and implementation of machine learning-based policy.

The line on the lower bound of the set in Fig. 9 represents the changes in antibiotic use and treated UTIs for policies that do not delegate any decisions to physicians. Full automation is inferior throughout the risk range which generalizes the findings in Section 6.1.

## Appendix F Efficiency and group fairness

The policies we consider are redistributive, following much of the literature (Kleinberg et al., 2015, 2018a; Hastings et al., 2020; Mullainathan & Obermeyer, 2022). Fairness concerns become salient, perhaps more so than for human biases, when machine learning predictions form the basis of decision outcomes (Kleinberg et al., 2018b; Cowgill & Tucker, 2019; Rambachan et al., 2020; Coston et al., 2021). A growing literature has pointed out that excluding sensitive predictors in pursuit of fairness can be detrimental to aggregate outcomes as well as to disadvantaged groups (Kleinberg et al., 2018b; Cowgill & Tucker, 2019; Manski et al, 2022). Hence, to cast light on potential fairness concerns and the cost of alleviating them, we assess and adapt our policy function on subgroups of patients but keep risk predictions unchanged.

We take a pragmatic approach and assess groups divided by age, gender, income, and immigration status. Fairness concerns are salient for these patient characteristics but they are also important predictors of UTI reported in Table 6 in Appendix A.5^1^. We first quantify redistribution between subgroups based on the main policy parameters reported in Section 6. Panel A in Tables 10 to 13 shows that antibiotic use decreases more strongly for young, male, immigrant, and high-income patients. All subgroups, except for income-based groups, deviate significantly from the aggregate outcome of the main policy. The main policy increases the number of treated UTI for women while lowering the number of treated UTIs for men, and fails to lower overall antibiotic use for women. Similarly, it reduces the number of treated UTI for patients with immigrant status. Hence, the main policy achieves reductions at the cost of discrepancies between patient subgroups and violates the constraint on the number of treated UTI.

To maintain the number of treated UTI for each group, we solve Eq. 6 separately by subgroup and evaluate policy outcomes. Panel B of Tables 10 to 13 reports group-specific and aggregate outcomes for policy parameters $k_L^j$ and $k_H^j$ optimized for patient group *j*. With this policy, reductions in antibiotic use are similar across groups with the exception that males have a larger reduction than women. Throughout, fewer physician decisions are overruled in aggregate and more decisions are delegated to physicians compared to the main policy. The group-specific policies reduce aggregate antibiotic use by 6.1 to 8.1 percent compared to the 8.1 percent reduction attained by the main policy, illustrating the trade-off between efficiency and group fairness.

**Table 10:** Counterfactual policy outcomes by age

	age < 48	48 $\le$ age	Aggregated
Panel A: Main algorithm			
$k_L$	0.320	0.320	0.320
$k_H$	0.601	0.601	0.601
Change in treated UTI, in %	$-19.9$	15.2	0.0
	$[-21.1, -18.7]$	[13.5, 16.7]	$[ -1.0, 1.0]$
Change in antibiotic use, in %	$$-28.7$$	10.7	$$ -8.1$$
	$[-29.6, -27.7]$	[9.4, 12.0]	$[ -8.9, -7.2]$
Change in overprescribing, in %	$-39.3$	2.3	$-20.3$
	$[-40.9, -37.6]$	[0.2, 4.8]	$[-21.7, -18.8]$
GP decisions overruled, in %	12.8	17.1	15.0
	[12.4, 13.2]	[16.6, 17.6]	[14.7, 15.3]
Patients delegated to GPs, in %	51.4	54.2	52.8
	[50.7, 52.0]	[53.6, 54.9]	[52.3, 53.3]
Panel B: Sub-group algorithm			
$k_L^j$	0.211	0.378	-
$k_H^j$	0.535	0.653	-
Change in treated UTI, in %	0.0	0.0	0.0
	$[ -1.0, 0.9]$	$[ -1.5, 1.5]$	$[ -1.0, 1.0]$
Change in antibiotic use, in %	$$ -4.8$$	$$ -7.2$$	$$ -6.1$$
	$[ -5.6, -4.0]$	$[ -8.5, -6.0]$	$[ -6.8, -5.3]$
Change in overprescribing, in %	$-10.7$	$-20.9$	$-15.3$
	$[-12.1, -9.1]$	$[-23.2, -18.7]$	$[-16.7, -14.0]$
GP decisions overruled, in %	6.2	18.6	12.4
	[5.9, 6.5]	[18.1, 19.1]	[12.1, 12.8]
Patients delegated to GPs, in %	74.0	48.2	61.0
	[73.4, 74.6]	[47.6, 48.9]	[60.6, 61.5]
Consultations	24, 047	24, 359	48, 406
Bacterial UTIs	7, 744	11, 071	18, 815
Treated UTIs	4, 935	6, 467	11, 402
Antibiotic prescriptions	9, 004	9, 868	18, 872
Overprescribing	4, 069	3, 401	7, 470

95% confidence intervals are based on 1000 bootstrap samples of 2011 and 2012 where machine learning predictions and the policy parameters $(k_{L},k_{H})$ and $(k_{L}^j,k_{H}^j)$ remain fixed

**Table 11:** Counterfactual policy outcomes by gender

	Female	Male	Aggregated
Panel A: Main algorithm			
$k_L$	0.320	0.320	0.320
$k_H$	0.601	0.601	0.601
Change in treated UTI, in %	5.0	$-33.1$	0.0
	[4.0, 6.0]	$[-36.5, -29.8]$	$[ -1.0, 1.0]$
Change in antibiotic use, in %	0.6	$$-51.9$$	$$ -8.1$$
	$[ -0.2, 1.4]$	$[-53.9, -49.8]$	$[ -8.9, -7.2]$
Change in overprescribing, in %	$$ -6.9$$	$$-69.3$$	$$-20.3$$
	$[ -8.3, -5.4]$	$[-71.7, -66.8]$	$[-21.7, -18.8]$
GP decisions overruled, in %	13.4	20.0	15.0
	[13.0, 13.7]	[19.3, 20.7]	[14.7, 15.3]
Patients delegated to GPs, in %	63.5	18.1	52.8
	[63.0, 64.1]	[17.4, 18.8]	[52.3, 53.3]
Panel B: Sub-group algorithm			
$k_L^j$	0.326	0.207	-
$k_H^j$	0.650	0.520	-
Change in treated UTI, in %	0.0	0.0	0.0
	$[ -1.0, 0.9]$	$[ -3.4, 3.5]$	$[ -1.0, 0.9]$
Change in antibiotic use, in %	$$ -5.4$$	$$-20.4$$	$$ -7.9$$
	$[ -6.2, -4.6]$	$[-22.5, -18.2]$	$[ -8.7, -7.1]$
Change in overprescribing, in %	$-14.5$	$-39.4$	$-19.9$
	$[-15.8, -13.0]$	$[-42.4, -36.2]$	$[-21.1, -18.6]$
GP decisions overruled, in %	12.2	15.2	12.9
	[11.9, 12.5]	[14.5, 15.8]	[12.6, 13.2]
Patients delegated to GPs, in %	65.5	37.1	58.8
	[65.0, 66.0]	[36.2, 38.0]	[58.3, 59.2]
Consultations	36, 960	11, 446	48, 406
Bacterial UTIs	16, 101	2, 714	18, 815
Treated UTIs	9, 905	1, 497	11, 402
Antibiotic prescriptions	15, 761	3, 111	18, 872
Overprescribing	5, 856	1, 614	7, 470

95% confidence intervals are based on 1000 bootstrap samples of 2011 and 2012 where machine learning predictions and the policy parameters $(k_{L},k_{H})$ and $(k_{L}^j,k_{H}^j)$ remain fixed

**Table 12:** Counterfactual policy outcomes by immigration status

	Immigrant	Non-immigrant	Aggregated
Panel A: Main algorithm			
$k_L$	0.320	0.320	0.320
$k_H$	0.601	0.601	0.601
Change in treated UTI, in %	$-33.7$	4.4	0.0
	$[-36.7, -30.7]$	[3.4, 5.5]	$[ -1.0, 1.0]$
Change in antibiotic use, in %	$$-46.9$$	$$ -1.5$$	$$ -8.1$$
	$[-48.9, -44.8]$	$[ -2.5, -0.6]$	$[ -8.9, -7.2]$
Change in overprescribing, in %	$-59.5$	$-11.4$	$-20.3$
	$[-62.2, -56.6]$	$[-13.0, -9.9]$	$[-21.7, -18.8]$
GP decisions overruled, in %	20.0	14.0	15.0
	[19.2, 20.8]	[13.7, 14.3]	[14.7, 15.3]
Patients delegated to GPs, in %	32.8	56.7	52.8
	[31.8, 33.9]	[56.2, 57.3]	[52.3, 53.3]
Panel B: Sub-group algorithm			
$k_L^j$	0.245	0.332	-
$k_H^j$	0.459	0.627	-
Change in treated UTI, in %	0.0	0.0	0.0
	$[ -3.1, 3.1]$	$[ -1.1, 1.0]$	$[ -1.1, 1.0]$
Change in antibiotic use, in %	$$ -9.2$$	$$ -6.6$$	$$ -7.0$$
	$[-11.4, -6.9]$	$[ -7.5, -5.7]$	$[ -7.8, -6.2]$
Change in overprescribing, in %	$-18.0$	$-17.6$	$-17.7$
	$[-21.5, -14.5]$	$[-19.1, -16.1]$	$[-19.0, -16.3]$
GP decisions overruled, in %	14.0	13.9	13.9
	[13.2, 14.7]	[13.6, 14.3]	[13.6, 14.3]
Patients delegated to GPs, in %	53.8	55.8	55.5
	[52.8, 55.0]	[55.3, 56.3]	[55.0, 55.9]
Consultations	7, 934	40, 472	48, 406
Bacterial UTIs	2, 269	16, 546	18, 815
Treated UTIs	1, 322	10, 080	11, 402
Antibiotic prescriptions	2, 708	16, 164	18, 872
Overprescribing	1, 386	6, 084	7, 470

95% confidence intervals are based on 1000 bootstrap samples of 2011 and 2012 where machine learning predictions and the policy parameters $(k_{L},k_{H})$ and $(k_{L}^j,k_{H}^j)$ remain fixed

**Table 13:** Counterfactual policy outcomes by income

	Income < 175.000	175.000 $\le$ Income	Aggregated
Panel A: Main algorithm			
$k_L$	0.320	0.320	0.320
$k_H$	0.601	0.601	0.601
Change in treated UTI, in %	1.2	$$ -1.1$$	0.0
	$[ -0.4, 2.7]$	$[ -2.5, 0.3]$	$[ -1.0, 1.0]$
Change in antibiotic use, in %	$$ -6.7$$	$$ -9.4$$	$$ -8.1$$
	$[ -7.9, -5.3]$	$[-10.4, -8.3]$	$[ -8.9, -7.2]$
Change in overprescribing, in %	$-19.0$	$-21.5$	$-20.3$
	$[-21.1, -16.4]$	$[-23.3, -19.7]$	$[-21.7, -18.8]$
GP decisions overruled, in %	15.6	14.4	15.0
	[15.1, 16.0]	[13.9, 14.8]	[14.7, 15.3]
Patients delegated to GPs, in %	49.7	56.0	52.8
	[49.1, 50.4]	[55.3, 56.6]	[52.3, 53.3]
Panel B: Sub-group algorithm			
$k_L^j$	0.326	0.303	-
$k_H^j$	0.601	0.626	-
Change in treated UTI, in %	0.0	0.0	0.0
	$[ -1.5, 1.6]$	$[ -1.3, 1.3]$	$[ -1.0, 1.0]$
Change in antibiotic use, in %	$$ -8.1$$	$$ -8.1$$	$$ -8.1$$
	$[ -9.4, -6.8]$	$[ -9.1, -7.0]$	$[ -8.9, -7.2]$
Change in overprescribing, in %	$-21.0$	$-20.1$	$-20.5$
	$[-23.0, -18.7]$	$[-21.7, -18.3]$	$[-21.7, -19.1]$
GP decisions overruled, in %	16.2	12.3	14.3
	[15.7, 16.6]	[11.9, 12.7]	[14.0, 14.6]
Patients delegated to GPs, in %	48.1	61.1	54.5
	[47.6, 48.8]	[60.5, 61.8]	[54.1, 55.0]
Consultations	24, 603	23, 803	48, 406
Bacterial UTIs	9, 728	9, 087	18, 815
Treated UTIs	5, 576	5, 826	11, 402
Antibiotic prescriptions	9, 108	9, 764	18, 872
Overprescribing	3, 532	3, 938	7, 470

95% confidence intervals are based on 1000 bootstrap samples of 2011 and 2012 where machine learning predictions and the policy parameters $(k_{L},k_{H})$ and $(k_{L}^j,k_{H}^j)$ remain fixed

## Appendix G Waiting for molecule-specific resistance information

**Table 14:** Antibiotic resistance for positive E. coli test results conditional on *d* and $k_H \le m(x)$

	$d=0, \; k_H \le m(x)$		$d=1, \; k_H \le m(x)$		
Antibiotic (ATC-code)	Obs	Resistance	Obs	Resistance	Difference
Ampicillin (J01CA01)	1,237	0.437	1,907	0.390	0.047 [0.012 ,0.083]
Mecillinam (J01CA11)	1,237	0.058	1,907	0.036	0.023 [0.007 ,0.038]
Trimethoprim (J01EA01)	1,237	0.310	1,907	0.261	0.050 [0.017 ,0.082]
Sulfamethizole (J01EB02)	1,237	0.373	1,907	0.331	0.042 [0.007 ,0.077]
Ciprofloxacin (J01MA02)	1,237	0.089	1,907	0.056	0.033 [-0.003 ,0.068]
Nitrofurantion (J01XE01)	1,237	0.042	1,907	0.027	0.015 [0.002 ,0.029]

Table 14 shows resistance rates for E. coli bacteria for patients with predicted risk *m*(*x*) larger than threshold $k_H$, conditional on physician treatment decisions and a positive E.coli test result. We find small differences in resistance against most antibiotics prescribed for UTI. When physicians treat instantly and bacteria are found, these have one to five percentage points lower resistance levels than when physicians decide to wait and bacteria are found

## Footnotes

1 Worldwide, 4.95 million deaths are estimated to be associated with antibiotic resistance and 1.27 million deaths are directly attributable (Murray et al., 2022; Laxminarayan, 2022). In the US alone, antibiotic-resistant infections are estimated to cause $20 billion in direct healthcare costs and $35 billion in lost productivity each year (CDC, 2013).

2 See *Medicinrådets behandlingsvejledning vedrørende urinvejsinfektioner* (https://medicinraadet.dk/media/ucsjy4e4/medicinrådets-behandlingsvejledning-vedr-urinvejsinfektioner-vers-1-1_adlegacy.pdf) or *Urinary Tract Infections* (https://www.mayoclinic.org/diseases-conditions/urinary-tract-infection/symptoms-causes/syc-20353447) by the Cleveland Clinic, accessed 11/2/2022.

3 Less frequently used antibiotics are nitrofurantoin, trimethoprim, amoxicillin, fluoroquinolones, and fosfomycin.

4 See Thornley et al. (2020) and https://www.bbc.com/news/uk-england-derbyshire-49031625, accessed on 12/7/2022. In Denmark, pivmecillinam is the recommended first-line antibiotic while nitrofurantoin or sulfamethizole should be given where penicillin is clinically unsuitable. See UTI guidelines by the Danish Medical Council at https://medicinraadet.dk/anbefalinger-og-vejledninger/behandlingsvejledninger/urinvejsinfektioner-uvi , accessed on 12/5/2022.

5 See https://medicinraadet.dk/media/ucsjy4e4/medicinrådets-behandlingsvejledning-vedr-urinvejsinfektioner-vers-1-1_adlegacy.pdf, accessed 11/2/2022.

6 We only observe the purchase date of a prescription which might differ from the date the physician provided the patient with the prescription. Hence, we must define what constitutes an initial prescription and choose to do so based on the patient purchasing the antibiotic on the day of the test or the following day. Defining initial prescriptions as any antibiotic purchased between the test date and the date the laboratory answer is provided to the physician does not qualitatively change the result of our analysis. We choose the shorter definition of an initial prescription for our main analysis as we want to exclude potential prescriptions that result from unobserved additional contact between the patient and the physician while awaiting the test result.

7 Out of the 1,557 covariates 1,038 are categorical variables that are transformed into dummy variables for each category. The final number of covariates for XGBoost is 12,727.

8 The optimal tuning parameter lambda is 0.0087 on the hyperparameter folds.

9 The sample is drawn such that it has the same number of observations as the analysis sample for $y=0$.

10 The sample is drawn such that it has the same number of observations as the analysis sample for $y=1$.

11 An alternative payoff function that includes the potential social cost of a follow-up prescription to a patient who suffers from a bacterial UTI but did not receive antibiotic treatment at the initial consultation has the following form:

$$\begin{aligned} \tilde{\pi }(d; y)&= - \alpha y(1-d) - \beta d - \beta (1-\rho ) y(1-d) \\&= - (\alpha + \beta (1-\rho ))y(1-d) - \beta d \\&= - \tilde{\alpha } y(1-d) - \beta d , \end{aligned}$$

where $d \in (0,1)$ is the prescription decision at the initial consultation, $y \in (0,1)$ is the sickness state and $\rho \in (0,1)$ is the spontaneous natural recovery rate that occurs while the patient awaits the test result. We obtain an expression similar to Eq. 1 with the only difference being the interpretation of the sickness cost.

12 This specific policy objective also minimizes overprescribing since the change in prescribing to non-UTI cases can be written generally as

$$\begin{aligned} \sum _{i \in \mathcal {I}} \delta _i(1-y_i) - \sum _{i \in \mathcal {I}} d_i(1-y_i)&= \sum _{i \in \mathcal {I}} (\delta _i-d_i) - \sum _{i \in \mathcal {I}} y_i(\delta _i - d_i), \end{aligned}$$

which equals the change in antibiotic use when the change in treated UTI, the last term above, is zero.

13 An alternative policy could include the physician’s decision as a predictor in the machine learning algorithm combined with prescription rules using a single threshold. While allowing more flexible combinations of physician decisions with other variables in the prediction algorithm, such an implementation would involve higher physician effort because her decision would be a required input at every consultation. Huang et al. (2022) use such a rule and find results similar to the policy we consider.

14 Similar policy outcomes are obtained using LASSO predictions as reported in Table 7 in Appendix C. The lower prediction quality achieved by LASSO results in an increase in antibiotic use by 11.3 percent and an increase in overprescribing by 28.7, while the share of overruled physician decisions of 41.2 is close to the XGBoost results.

15 Table 8 in Appendix C reports results based on LASSO predictions, demonstrating similar policy outcomes. The slightly lower prediction quality achieved by LASSO results in an almost ten percentage points larger share of decisions delegated to physicians and a smaller overall reduction in antibiotic use at 7.0 percent. Our qualitative findings do not appear to depend on the choice of prediction algorithm.

16 An additional concern might be omitted-payoff bias (Kleinberg et al., 2018a). First, the policy may give antibiotics to high-risk patients who would not be treated even with a certain presence of bacteria. Of all patients with positive test results to whom the policy assigns an antibiotic but physicians did not, 71.8 percent receive an antibiotic after the test result is known. Given an estimated spontaneous recovery rate of 24 percent (Ferry et al., 2004), decisions implemented by the policy resemble physician choices under full information. Second, physicians may wait for antibiotic resistance information before prescribing. For E. coli bacteria, the predominant cause of UTI, Table 14 in Appendix G shows small differences in resistance for high-risk patients, conditional on the decision to prescribe or wait. Quantifying the benefits of resistance predictions in primary care is a promising avenue for further research.

17 Even though we do not directly observe UTI consultations when no laboratory test was made, we can approximate them by observing claimed rapid diagnostic tests, with at least one being utilized in nearly all initial UTI consultations.
